# Chronic myeloproliferative neoplasms with concomitant *CALR* mutation and *BCR::ABL1* translocation: diagnostic and therapeutic implications of a rare hybrid disease

**DOI:** 10.3389/fcell.2024.1391078

**Published:** 2024-03-26

**Authors:** Magda Zanelli, Valentina Fragliasso, Giuseppe Gaetano Loscocco, Francesca Sanguedolce, Giuseppe Broggi, Maurizio Zizzo, Andrea Palicelli, Stefano Ricci, Elisa Ambrogi, Giovanni Martino, Sara Aversa, Francesca Coppa, Pietro Gentile, Fabrizio Gozzi, Rosario Caltabiano, Nektarios Koufopoulos, Aleksandra Asaturova, Luca Cimino, Alberto Cavazza, Giulio Fraternali Orcioni, Stefano Ascani

**Affiliations:** ^1^ Pathology Unit, Azienda USL-IRCCS di Reggio Emilia, Reggio Emilia, Italy; ^2^ Laboratory of Translational Research, Azienda USL-IRCCS di Reggio Emilia, Reggio Emila, Italy; ^3^ Department of Experimental and Clinical Medicine, CRIMM, Center of Research and Innovation of Myeloproliferative Neoplasms, Azienda Ospedaliero-Universitaria Careggi, University of Florence, Florence, Italy; ^4^ Doctorate School GenOMec, University of Siena, Siena, Italy; ^5^ Pathology Unit, Policlinico Riuniti, University of Foggia, Foggia, Italy; ^6^ Department of Medical and Surgical Sciences and Advanced Technologies “G.F. Ingrassia” Anatomic Pathology, University of Catania, Catania, Italy; ^7^ Surgical Oncology Unit, Azienda USL-IRCCS di Reggio Emilia, Reggio Emilia, Italy; ^8^ Pathology Unit, Azienda Ospedaliera Santa Maria di Terni, University of Perugia, Terni, Italy; ^9^ Hematology, Centro di Ricerca Emato-Oncologica-C.R.E.O., University of Perugia, Perugia, Italy; ^10^ Ocular Immunology Unit, Azienda USL-IRCCS di Reggio Emilia, Reggio Emilia, Italy; ^11^ Second Department of Pathology, Medical School, National and Kapodistrian University of Athens, Attikon University Hospital, Athens, Greece; ^12^ Pathology Department, FSBI “National Medical Research Centre for Ostetrics, Gynecology and Perinatology Named After Academician V.I Kulakov” of the Ministry of Health of the Russian Federation, Moscow, Russia; ^13^ Department of Surgery, Medicine, Dentistry and Morphological Sciences, University of Modena and Reggio Emilia, Modena, Italy; ^14^ Pathology Unit, Azienda Ospedaliera di Cuneo, Cuneo, Italy

**Keywords:** BCR::ABL1, CALR, chronic myeloid leukemia, myeloproliferative neoplasm, primary myelofibrosis, essential thrombocythemia

## Abstract

Myeloproliferative neoplasms (MPNs) are subdivided into Philadelphia (Ph) chromosome-positive chronic myeloid leukemia (CML) and Ph-negative MPNs. *BCR::ABL1* translocation is essential for the development and diagnosis of CML; on the other hand, the majority of Ph-negative MPNs are characterized by generally mutually exclusive mutations of Janus kinase 2 (*JAK2*), calreticulin (*CALR*), or thrombopoietin receptor/myeloproliferative leukemia (*MPL*). *CALR* mutations have been described essentially in *JAK2* and *MPL* wild-type essential thrombocythemia and primary myelofibrosis. Rarely coexisting *CALR* and *MPL* mutations have been found in Ph-negative MPNs. *BCR::ABL1* translocation and *JAK2* mutations were initially considered mutually exclusive genomic events, but a discrete number of cases with the combination of these genetic alterations have been reported. The presence of *BCR::ABL1* translocation with a coexisting *CALR* mutation is even more uncommon. Herein, starting from a routinely diagnosed case of *CALR*-mutated primary myelofibrosis subsequently acquiring *BCR::ABL1* translocation, we performed a comprehensive review of the literature, discussing the clinicopathologic and molecular features, as well as the outcome and treatment of cases with *BCR::ABL1* and *CALR* co-occurrence.

## 1 Introduction

Myeloproliferative neoplasms (MPNs) are clonal disorders with proliferation of at least one hematopoietic lineage and are subdivided into Philadelphia (Ph)-positive and Ph-negative forms (WHO, 2017; Arber et al., 2022; Khoury et al., 2022). Ph-positive chronic myeloid leukemia (CML) is typically characterized by the presence of the pathognomonic Ph-chromosome with translocation t (9; 22), resulting in the *BCR::ABL1* oncogene. In 2005, it was discovered that the activating mutation in Janus kinase 2 (*JAK2*), mostly at codon 617 (*JAK2 V617F*), is involved in the development of Ph-negative MPNs, including polycythemia vera (PV), essential thrombocythemia (ET), and primary myelofibrosis (PMF) (Cazzola and Kralovics, 2014; WHO, 2017; Arber et al., 2022; Khoury et al., 2022). Subsequently, novel insights into Ph-negative MPN development were provided by the identification of the activating mutations in the myeloproliferative leukemia (*MPL*) gene in 2006 and *CALR* gene in 2013 (Klampfl et al., 2013; Nangalia et al., 2013; Rumi et al., 2014a). *JAK2* mutations occur in 95% of PV and in 50%–60% of ET and PMF. After *JAK2* mutations, *CALR* mutations are the second most common driver mutations in ET and PMF, being reported primarily in the context of *JAK2* and MPL wild-type PMF (25%–35%) and ET (20%–25%) (Rumi et al., 2014b; Tefferi et al., 2014). *MPL* mutation occurs in 5%–10% of ET and PMF.

*CALR* mutations activate the *JAK/STAT* pathway through *MPL*, causing a high platelet (PLT) count. Although approximately 50 different *CALR* mutations have been reported, the most common *CALR* mutations are type 1 with a 52-base pair deletion and type 2 with a 5-base pair insertion (Rumi et al., 2014b; Tefferi et al., 2014; Pietra et al., 2016; Loscocco et al., 2021; Guglielmelli et al., 2024; Loscocco et al., 2024). *CALR* type 1 mutation has been associated with an increased risk of myelofibrotic transformation in ET patients; on the other hand, *CALR* type 2 mutation is identified in ET with an indolent behavior and low thrombotic risk despite an elevated platelet (PTL) count (Rumi et al., 2014b; Tefferi et al., 2014; Pietra et al., 2016). *CALR* type 1 and *CALR* type 2 mutations show different morphological features and different outcomes. Both 2022 WHO classification and ICC classification take into consideration these molecular findings, refining the current diagnostic criteria of Ph-negative MPNs, but neither classification addresses the issue of MPNs, presenting more than one “driver” genetic alteration (Arber et al., 2022; Khoury et al., 2022).

*BCR::ABL1* rearrangement and *JAK2/MPL/CALR* mutations were initially considered mutually exclusive genetic events. However, despite being a rare occurrence, a number of cases with concomitant Ph-positive and *JAK2-*mutated MPNs have been described with a frequency ranging from 0.2% to 2.5%, depending on different studies (Pieri et al., 2011; Martin-Cabrera et al., 2017; Soderquist et al., 2018).

The association of *CALR* mutations with *BCR::ABL1* rearrangement is much rarer, being reported in isolated cases. In this article, we described a case from our daily practice with a long history of *CALR*-mutated PMF, subsequently developing CML with gaining of *BCR::ABL1* rearrangement; meanwhile, through a literature search, we identified 23 cases harboring both *CALR* mutation and *BCR::ABL1* rearrangement (Bonzheim et al., 2015; Cabagnols et al., 2015; Loghavi et al., 2015; Diamond et al., 2016; Seghatoleslami et al., 2016; Dogliotti et al., 2017; Gilles et al., 2017; Kandarpa et al., 2017; Blouet et al., 2018; Boddu et al., 2018; De Roeck et al., 2018; Klairmont et al., 2018; Lewandowski et al., 2018; Xia et al., 2019; Balducci et al., 2020; Da Costa et al., 2020; Guidotti et al., 2020; Liu et al., 2020; Yoon et al., 2020; Sobieralski et al., 2022; Huo et al., 2023).

Clinical manifestations, pathological features, clonal findings, and management of these cases with the concomitant *CALR* mutation and *BCR::ABL1* are discussed.

In cases with discordant clinical and molecular features or uncommon BM histology, the co-occurrence of *BCR::ABL1*-positive CML with another Ph-negative MPN should be suspected.

## 2 Explicative case from routine daily practice

**FIGURE 1:**
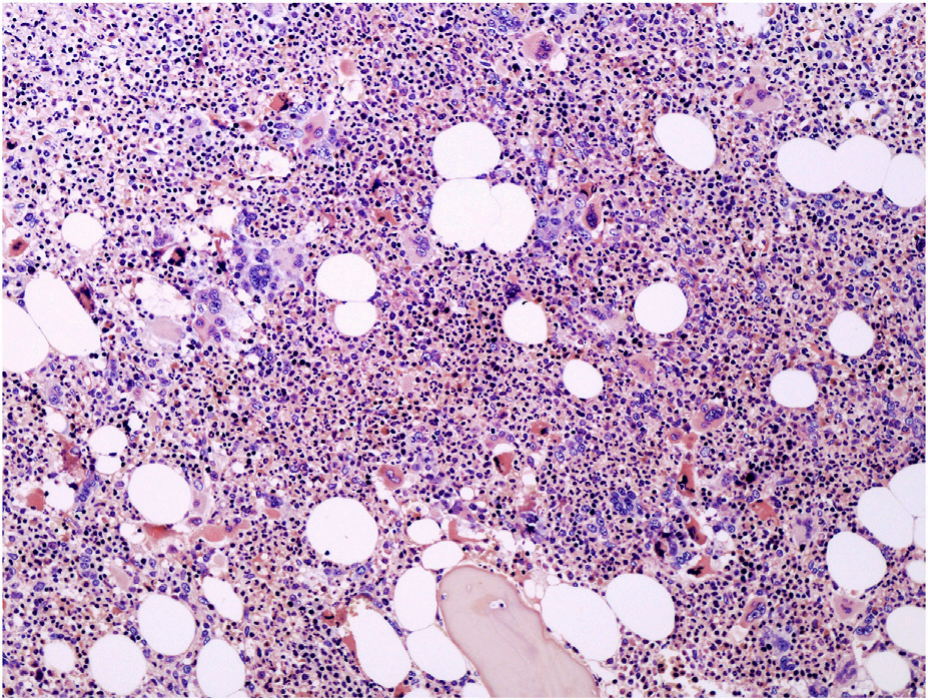
Medium power view of BM biopsy showing a hypercellular marrow with the prevalence of granulopoiesis and clustering of variably sized atypical MKs (hematoxylin and eosin, ×100 magnification).

**FIGURE 2:**
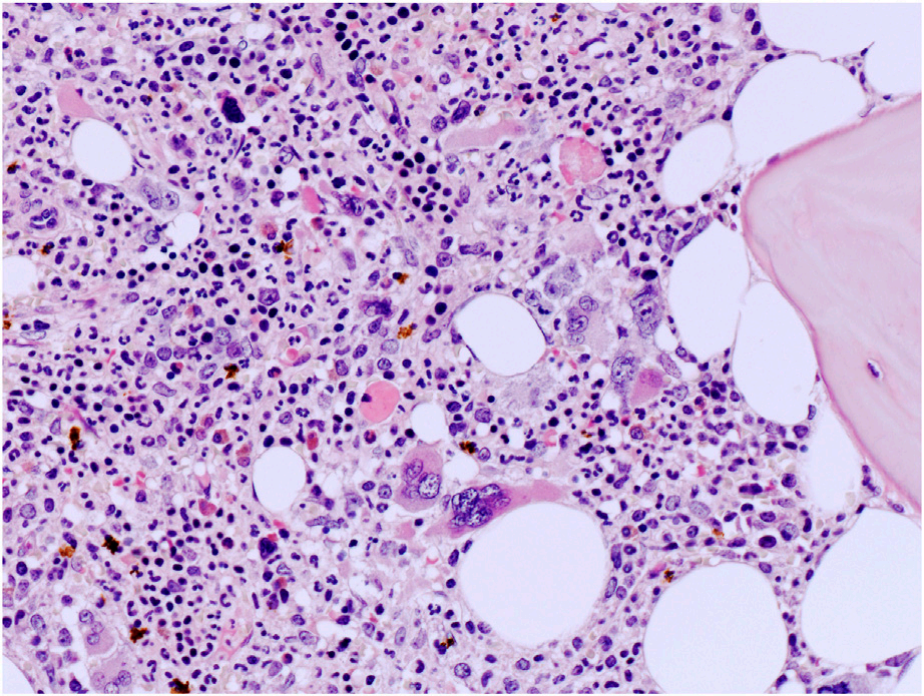
High power view of BM biopsy highlighting atypical MKs with non-classical CML features such as large MKs with bulbous nuclei (hematoxylin and eosin, ×200 magnification).

A 45-year-old man was noted to have a new onset thrombocytosis with a PTL count of 526 × 10^9^/L and normochromic, normocytic mild anemia (Hb 10.5 g/dL), whereas the white blood cell (WBC) count (8.02 × 10^9^/L) was within the normal limit with 63% neutrophils, 27% lymphocytes, and 8% monocytes. A moderate splenomegaly was present. Bone marrow (BM) biopsy showed a slightly hypercellular marrow with the prevalence of a normally maturing myeloid lineage; the erythroid lineage in different stages of maturation was reduced; and the megakaryocytic lineage was expanded with even dense clustering of variably sized elements, including megakaryocytes with hyperchromatic nuclei. CD34-positive hematopoietic precursors were not increased. Grade 0 reticulin fibrosis was present. Molecular studies showed wild-type *JAK2 V617F* and *MPL* genes, whereas *CALR* exon 9 mutation type 1 (60% allelic burden) was identified. No *BCR::ABL1* rearrangement was found. Karyotype analysis was not available. The combination of clinical, pathologic, and molecular features was in keeping with the diagnosis of MPN and suggestive of PMF, in the pre-fibrotic stage. Anagrelide was initially administered, followed by hydroxyurea (HU) 3 years later with a good hematological control of the disease. After 11 years, a progressive increase in the WBC count was noted (57 × 10^9^/L), with mild anemia (Hb 9.2 g/dL) and normal PTL count (177 × 10^9^/L). Splenomegaly (18 cm diameter) was detected. BM biopsy showed a markedly hypercellular marrow (95% cellularity) with prevalent and normally maturing granulopoiesis, the erythroid lineage was reduced with features of dyserythropoiesis, and megakaryocytes were increased in number with large-sized elements along with small-sized cells with hyperchromatic nuclei and evident dense clustering (Figures 1, 2). Grade II reticulin fibrosis was present. Repeated molecular studies confirmed *CALR* exon 9 mutation (allelic burden 40%) and *JAK2 V617F* and *MPL* negativity, whereas the *BCR::ABL1* rearrangement was identified. RT-PCR identified the *BCR-ABL1* fusion transcript of the p210 variant (allelic burden 88%). Karyotype analysis detected a 46XY karyotype with t (9:22) (q34; q11) translocation. Altogether, the clinicopathologic and molecular findings supported the diagnosis of PMF (with *CALR* mutation) and subsequent occurrence of the *BCR::ABL1* rearrangement, therefore, interpretable as the coexistence of a Ph-negative MPN with CML. Tyrosine-kinase therapy (TKI) with nilotinib (600 mg/die) associated with ruxolitinib was given. After introduction of TKI treatment, the patient achieved a deep molecular response (DMR) within 6 months, with negativity of *BCR-ABL1* but persistence of CALR type 1 mutation (allelic burden 45%). The patient is still on combined treatment (ruxolitinib plus nilotinib) with DMR and good control of disease despite persistence of *CALR* mutation, 7 years after CML occurrence.

## 3 Systematic review of the literature: methods

We performed a systematic review adhering to the Preferred Reported Items for Systematic Reviews and Meta-Analyses (PRISMA) guidelines. The literature search was carried out through PubMed/MEDLINE, Embase, Scopus, Cochrane Library (Cochrane Database of Systematic Reviews), Cochrane Central Register of Controlled Trials (CENTRAL), and Web of Science (Science and Social Science Citation Index) databases, with the following non-MeSH/MeSH terms “*CALR*” AND *BCR::ABL1* concomitant” [Mesh]. The search was performed from 2013 when *CALR* mutations were identified to be MPN-driven mutations to January 2024. The criteria for inclusion were as follows: 1) MPNs with concomitant *CALR* mutation and *BCR::ABL1* translocation; 2) retrospective studies, case reports and/or case series, and literature review. The exclusion criteria were as follows: 1) studies not published in English; 2) lack of concomitant *CALR* mutation and *BCR::ABL1* translocation. The papers were identified by three independent reviewers (M. Zanelli, VF, and GGL), first considering title, abstract, and key words and then reading the article full texts to evaluate if the articles met the inclusion criteria. From selected papers, the following information was collected: author’s surname, year of publication, patient’s age and sex, first disease diagnosis, second disease diagnosis, interval between the first and second diseases, BM histology during the disease course, type of *CALR* mutation, interaction between *CALR* and *BCR::ABL1* clones, and treatment and prognosis. A third independent reviewer (AS) re-examined all collected results and resolved divergences.

## 4 Results of literature cases and the present case

### 4.1 Epidemiological and clinical data

Our literature search identified 21 articles reporting a total of 23 cases with concurrent *BCR::ABL1* translocation and *CALR* mutation. The clinical, pathologic, and molecular characteristics, as well as treatment and outcome data, of the cases are summed up in Supplementary Table S1 (Supplementary Material).

Including our case, a total of 24 cases with concomitant *BCR::ABL1* and *CALR* mutation have been described so far (Bonzheim et al., 2015; Cabagnols et al., 2015; Loghavi et al., 2015; Diamond et al., 2016; Seghatoleslami et al., 2016; Dogliotti et al., 2017; Gilles et al., 2017; Kandarpa et al., 2017; Blouet et al., 2018; Boddu et al., 2018; De Roeck et al., 2018; Klairmont et al., 2018; Lewandowski et al., 2018; Xia et al., 2019; Balducci et al., 2020; Da Costa et al., 2020; Guidotti et al., 2020; Liu et al., 2020; Yoon et al., 2020; Sobieralski et al., 2022; Huo et al., 2023).

Median age at the first diagnosis was 59.6 (range: 26–90), with a moderate prevalence in female individuals (F:14/24, 58.33%; M: 10/24, 41.66%).

In the majority of cases with concurrent *BCR::ABL1* translocation and *CALR* mutation, CML was the first diagnosis, followed by Ph-negative MPN (13/24; 54.16%) (Cabagnols et al., 2015; Loghavi et al., 2015; Diamond et al., 2016; Dogliotti et al., 2017; Gilles et al., 2017; Kandarpa et al., 2017; Blouet et al., 2018; Lewandowski et al., 2018; Balducci et al., 2020; Da Costa et al., 2020; Guidotti et al., 2020; Yoon et al., 2020; Huo et al., 2023), and the Ph-negative MPNs were distributed as follows: four myeloproliferative neoplasm not otherwise specified (MPN, NOS); four PMF; and five ET.

In 9/24 cases (37.5%), CML was the second diagnosis preceded by Ph-negative MPNs (Bonzheim et al., 2015; Kandarpa et al., 2017; Boddu et al., 2018; De Roeck et al., 2018; Klairmont et al., 2018; Liu et al., 2020; Sobieralski et al., 2022), represented by four ET; three PMF; one MPN, NOS; and one post-essential thrombocythemia myelofibrosis (PET-MF).

In 2/24 cases (8.33%), Ph-negative MPN and CML occurred simultaneously (Seghatoleslami et al., 2016; Boddu et al., 2018), and the Ph-negative MPNs were one MPN, NOS and one PMF.

The median interval between the first and second diagnoses was 50 months (range: 3–154) in cases with CML preceding Ph-negative MPN and 141 months (range: 30–468) in cases with Ph-negative MPN preceding CML.

CML was found to be clinically in the chronic phase (CP) in all cases with the exception of one case with concomitant CML and MPN, NOS, in which CML was in the blast phase (BP) (Seghatoleslami et al., 2016).

In the group with CML preceding Ph-negative MPN, the clinical manifestations at the second disease occurrence were as follows: high PTL in 9/13 despite good molecular response of CML (Cabagnols et al., 2015; Dogliotti et al., 2017; Gilles et al., 2017; Lewandowski et al., 2018; Balducci et al., 2020; Da Costa et al., 2020; Guidotti et al., 2020; Yoon et al., 2020), increasing splenomegaly in 3/13 despite good molecular response of CML (Diamond et al., 2016; Kandarpa et al., 2017; Huo et al., 2023), anemia in 2/13 despite a good molecular response of CML (Cabagnols et al., 2015; Diamond et al., 2016), and appetite loss and fatigue in 1/13 despite a good molecular response of CML (Huo et al., 2023).

In the group with CML emerging as the second disease after Ph-negative MPN, the clinical manifestations at CML occurrence were as follows: high WBC in 9/9 (Bonzheim et al., 2015; Kandarpa et al., 2017; Boddu et al., 2018; De Roeck et al., 2018; Klairmont et al., 2018; Xia et al., 2019; Liu et al., 2020; Sobieralski et al., 2022), anemia in 3/9 (Kandarpa et al., 2017; Boddu et al., 2018; Xia et al., 2019), high PTL 1/9 (De Roeck et al., 2018), low PTL in 1/9 (Boddu et al., 2018), increasing splenomegaly in 5/9 (Kandarpa et al., 2017; Boddu et al., 2018; Liu et al., 2020; Sobieralski et al., 2022), hepatomegaly in 1/9 (Boddu et al., 2018), and fatigue and abdominal pain in 1/9 (De Roeck et al., 2018).

### 4.2 Histological features

In the group with CML preceding Ph-negative MPN (13/24 cases), BM histology at CML presentation in 10/13 cases with available BM data was as follows: CML (CP) histology in 2/10 (Kandarpa et al., 2017; Balducci et al., 2020), CML + fibrosis + hybrid MKs including both classical CML MKs with small and hypolobate nuclei and large hyperlobated forms in 3/10 (Loghavi et al., 2015; Gilles et al., 2017; Guidotti et al., 2020), CML + atypical MKs in 2/10 (Blouet et al., 2018; Da Costa et al., 2020), CML + fibrosis in 2/10 (Diamond et al., 2016; Yoon et al., 2020), and grade 2 reticulin fibrosis with no other BM data in 1/10 (Cabagnols et al., 2015). In this group, the emergence of the Ph-negative MPN phenotype usually occurred after TKI treatment and CML remission; the histology of BM at Ph-negative MPN emergence was available in 9/13 cases, and it was consistent with either PMF (4/9) (Loghavi et al., 2015; Diamond et al., 2016; Kandarpa et al., 2017; Huo et al., 2023), ET (4/9) (Dogliotti et al., 2017; Blouet et al., 2018; Balducci et al., 2020; Da Costa et al., 2020), or MPN, NOS (1/9) [ 25].

In the group with Ph-negative MPN preceding CML (9/24 cases), at presentation of first disease (Ph-negative MPN), BM histology was available in 6/9 cases, and it was consistent with either PMF (3/6) (Boddu et al., 2018; Sobieralski et al., 2022), ET (2/6) (Bonzheim et al., 2015; Liu et al., 2020), and MPN, NOS (1/6) (Klairmont et al., 2018). In this group, at the occurrence of CML, BM histological features were available in 7/9 cases as follows: CML (CP) histology in 1/7 (Bonzheim et al., 2015), CML + fibrosis in 3/7 (Klairmont et al., 2018; Liu et al., 2020; Sobieralski et al., 2022), and CML + fibrosis + hybrid MKs in 3/7 (Boddu et al., 2018; Xia et al., 2019).

Of 2/24 cases with concomitant CML and Ph-negative MPN, BM biopsy, performed only in 1/2 (CML + PMF), was obtained only at CML complete hematologic response and showed histological features consistent with PMF (Boddu et al., 2018).

### 4.3 Molecular data

In the group of CML preceding Ph-negative MPN (13/24 cases), data on the *CALR* type were available in 11/13 cases. *CALR* type 1 (52-bp deletion) was identified in 10/11 (Cabagnols et al., 2015; Loghavi et al., 2015; Diamond et al., 2016; Dogliotti et al., 2017; Gilles et al., 2017; Blouet et al., 2018; Balducci et al., 2020; Da Costa et al., 2020; Guidotti et al., 2020; Yoon et al., 2020) and *CALR* 34 bp deletion in 1/11 (Huo et al., 2023).

In the group of Ph-negative MPN preceding CML (9/24 cases), data on the *CALR* type were available in 8/9 cases, of which *CALR* type 1 was detected in 4/8 (Klairmont et al., 2018; Xia et al., 2019; Liu et al., 2020) and *CALR* type 2 (5-bp insertion) in 4/8 cases (Bonzheim et al., 2015; Boddu et al., 2018; De Roeck et al., 2018; Sobieralski et al., 2022). In the two cases of concomitant CML and Ph-negative MPN, *CALR* type 1 was found (Seghatoleslami et al., 2016; Boddu et al., 2018).

In the group of CML preceding Ph-negative MPN, *CALR* mutation and *BCR::ABL1* rearrangement were identified simultaneously at CML diagnosis in 10/10 cases, in which *CALR* was evaluated at initial CML diagnosis (Cabagnols et al., 2015; Loghavi et al., 2015; Diamond et al., 2016; Dogliotti et al., 2017; Gilles et al., 2017; Blouet et al., 2018; Lewandowski et al., 2018; Balducci et al., 2020; Yoon et al., 2020; Huo et al., 2023). Data on the interaction between the *BCR::ABL1* clone and *CALR* clone were available in 10/13 cases of this group; the Ph-positive clone resulted sensitive to TKI treatment with the progressive *BCR::ABL1* decrease, whereas the *CALR*-mutant clone was persistent in all 10 cases with *CALR* allelic burden increasing at *BCR::ABL1* decrease (Cabagnols et al., 2015; Loghavi et al., 2015; Diamond et al., 2016; Dogliotti et al., 2017; Gilles et al., 2017; Blouet et al., 2018; Lewandowski et al., 2018; Balducci et al., 2020; Yoon et al., 2020; Huo et al., 2023).

In the group of Ph-negative MPN preceding CML (9/24 cases), *CALR* was evaluated and found positive at initial Ph-negative MPN diagnosis only in 2/9 cases (Bonzheim et al., 2015), whereas in 7/9 cases, the *CALR* test was not performed at initial diagnosis because the test was not available. In all cases of this group, at CML diagnosis, both *CALR* mutation and *BCR::ABL1* rearrangement were detected (Bonzheim et al., 2015; Kandarpa et al., 2017; Boddu et al., 2018; De Roeck et al., 2018; Klairmont et al., 2018; Xia et al., 2019; Liu et al., 2020; Sobieralski et al., 2022). The interaction between *CALR* and *BCR::ABL1* clones was not available in 4/9 cases; the Ph-positive clone was sensitive to TKI and the *CALR* clone persistent in 4/9 cases (Bonzheim et al., 2015; Liu et al., 2020; Sobieralski et al., 2022), whereas in 1/9 cases, both *CALR* and *BCR::ABL1* clones persisted despite treatment with TKI, HU, and ruxolitinib (Boddu et al., 2018).

Of the two cases with concomitant CML and Ph-negative MPN, the interaction between the *CALR* and *BCR::ABL1* clone was available only in 1/2 cases, with the Ph-positive clone sensitive to TKI and the *CALR*-mutant clone persistent after DMR of CML (Boddu et al., 2018).

### 4.4 Treatment and prognosis

In the group of CML preceding Ph-negative MPN (13/24 cases), TKI treatment achieved DMR of CML in 10/13 cases (Loghavi et al., 2015; Diamond et al., 2016; Dogliotti et al., 2017; Kandarpa et al., 2017; Blouet et al., 2018; Balducci et al., 2020; Da Costa et al., 2020; Guidotti et al., 2020; Yoon et al., 2020; Huo et al., 2023), complete cytogenetic remission (CCyR) in 1/13 [ 23], and complete hematologic remission (CHR) in 1/13 (Gilles et al., 2017); however, in the 1/13 case, CML relapsed due to TKI stopping (Cabagnols et al., 2015). In 1/10 cases achieving DMR, TKI was stopped for gastric intolerance, and the patient was enrolled in a peptide CML vaccination protocol maintaining DMR of CML 12 years after TKI stopping (Dogliotti et al., 2017). Detailed data on different types of TKIs administered are present in Supplementary Table S1. In this group (CML preceding Ph-negative MPN), different treatments were administered in order to treat the Ph-negative MPN in 8/13 cases. In detail, HU in 1/8 (Yoon et al., 2020), interferon alpha (IFN) then replaced with HU and erythropoietin (EPO) in 1/8 (Cabagnols et al., 2015), HU followed by IFN + cytosine arabinoside (Ara-C) with slight PTL reduction in 1/8 (Lewandowski et al., 2018), HU followed by IFN in 1/8 (Dogliotti et al., 2017), hydroxycarbamide with a good control of ET in 1/8 (Blouet et al., 2018), hydroxycarbamide and acetylsalicylic acid (ASA) with good PTL control in 1/8 (Da Costa et al., 2020), HU with ASA with PTL normalization in 1/8 (Guidotti et al., 2020), and HU followed by anagrelide and subsequently by IFN with PTL normalization in 1/8 (Balducci et al., 2020). All patients of this group were alive at the last follow-up; OS was available in 12/13 cases with a median OS of 72 months (range 10–181).

In the group of Ph-negative MPN preceding CML (9/24 cases), TKI treatment achieved DMR in 4/7 cases with available follow-up data (De Roeck et al., 2018; Xia et al., 2019; Liu et al., 2020) and CHR in 2/7 cases (Bonzheim et al., 2015; Kandarpa et al., 2017), whereas in 1/7 cases, TKI did not achieve any response, and the patient underwent allogenic hematopoietic stem cell transplantation (allo-HSCT) with DMR (Boddu et al., 2018). In this group (Ph-negative MPN preceding CML), different therapies were administered for Ph-negative MPN as follows: IFN with no change in ET clinical features in 1/9 (Bonzheim et al., 2015); HU in 1/9 (Klairmont et al., 2018); anagrelide followed by ruxolitinib in 2/9 (Kandarpa et al., 2017); ASA in 1/9 (Xia et al., 2019); ruxolitinib with good control of ET in 1/9 (De Roeck et al., 2018); IFN + ASA with stable disease in 1/9 (Liu et al., 2020); ASA + HU, then ruxolitinib followed by spleen radiotherapy and allo-HSCT in 1/9 (Sobieralski et al., 2022); and HU, followed by ruxolitinib and subsequently by allo-HSCT in 1/9 with complete remission of both CML and Ph-negative MPN with undetectable genetic markers at 2 years from allo-HSCT (Boddu et al., 2018). All seven patients with available follow-up data were alive; median OS was 121 months (range 48–240).

Data on therapy and survival were available only in 1/2 cases with concomitant CML and Ph-negative MPN; in this case, TKI achieved DMR of CML, whereas no cytoreductive therapy was administered for Ph-negative MPN, and the patient was alive 24 months after diagnosis.

## 5 Discussion

The knowledge of the genetic basis of Ph-negative MPNs improved in the last few years because of the discovery of the main MPN driver mutations, including *JAK2*, *MPL*, and *CALR* mutations. The clinical manifestations and histological phenotype of MPNs mainly depend on their mutational status.

*BCR::ABL1* rearrangement, at the basis of CML development, and *JAK2* mutations were initially considered mutually exclusive genetic alterations. However, since *JAK2* first discovery in 2005, an increasing number of patients with concomitant *BCR::ABL1* rearrangement and *JAK2* mutations have been reported, and in a recent comprehensive review of the literature, we identified 87 cases carrying both genetic abnormalities (Zanelli et al., 2024).

First described in 2013, *CALR* mutations have been detected in the majority of *JAK2-* and *MPL*-negative ET and PMF. *CALR* and *BCR::ABL1* double-positive cases have been reported mainly as rare single-case descriptions. In the current review of the literature, we identified a total of 24 cases, including the present case, carrying the *CALR* mutation and *BCR::ABL1* rearrangement.

The coexistence of CML and Ph-negative MPN may show three possible scenarios: CML preceding Ph-negative MPN; CML developing in patients with a previous history of Ph-negative MPN; and, finally, simultaneous presentation of CML and Ph-negative MPN.

In our recent review analyzing *BCR::ABL1/JAK2 V617F* double-positive cases, the majority of patients fell into the group of *JAK2*-mutated MPN preceding CML (49.42%) (Zanelli et al., 2024).

Unlike *BCR::ABL1/JAK2* double-positive cases, the majority of cases carrying the *CALR* mutation and *BCR::ABL1* rearrangement fell into the group of CML preceding Ph-negative MPN (54.16%), followed by Ph-negative MPN preceding CML (37.5%) and, finally, by concomitant CML and Ph-negative MPN (8.33%). However, the number of *BCR::ABL1/CALR* double-positive cases reported so far is too small to draw any conclusion about the different frequencies of the three scenarios between *BCR::ABL1/JAK2* double-positive cases and patients carrying both *BCR::ABL1* and *CALR*.

The coexistence of the *BCR::ABL1* rearrangement and *CALR* mutation may change the clinical and laboratory manifestations of MPN and, for instance, in CML patients may be misinterpreted as failure of TKI therapy or disease transformation.

In CML patients, the persistence or occurrence of thrombocytosis, despite WBC decrease and good molecular *BCR::ABL1* response under TKI, should alert clinicians to perform molecular screening for Ph-negative MPN, including *JAK2*, *MPL*, and *CALR* mutations. Similarly, cases of Ph-negative MPN developing CML-like manifestations (WBC increase or splenomegaly), even years after clinical stability, should lead to additional molecular investigations to rule out CML occurrence.

The pathologist may suspect the coexistence of Ph-negative MPN and Ph-positive CML from a close examination of BM histology. Non-clustering small MKs, the so-called dwarf MKs, are normally found in CML, whereas large and clustered MKs are common in Ph-negative MPNs.

Therefore, the identification of large MKs with hyperlobulated nuclei in BM of CML patients should prompt additional genetic testing including *CALR* mutations to exclude the coexistence of a Ph-negative MPN. Some cases of our literature review showed unusual MK morphology with composite features (hybrid MKs with both small and large forms), which should represent a clue for molecular testing.

Of note, even in cases with CML preceding Ph-negative MPN, at CML diagnosis, BM histology was rarely that of classical CML in CP, but more frequently, CML histology was associated with either fibrosis or MKs, showing no clear-cut CML features or both fibrosis and hybrid MKs.

In the majority of cases in the group with CML preceding Ph-negative MPN, *CALR* mutation and *BCR::ABL1* translocation were retrospectively found to be coexistent at initial CML diagnosis, therefore explaining the abovementioned atypical histology at initial CML diagnosis.

The emergence of the *CALR*-mutated MPN phenotype often became clinically and histologically evident, following TKI therapy and CML remission as TKIs were generally ineffective for the *CALR*-mutated disease.

Data on the interaction between the *BCR::ABL1* clone and *CALR* clone commonly showed an inverse relation as *BCR::ABL1* decreased under TKI therapy, whereas the *CALR*-mutant clone persisted, often with a high allele burden, during the disease course despite successful TKI treatment of CML.

Similarly to *BCR::ABL1/JAK2* double-positive cases, even in patients carrying both *BCR::ABL1* and *CALR,* CML was easily managed with a good response to different types of TKIs. The *CALR*-mutated MPNs received different treatments (HU, anagrelide, IFN, and ruxolitinib) often as sequential therapies; however, data on the outcome are often incomplete and scarce; therefore, further studies are essential to establish the optimal management. In *BCR::ABL1/JAK2* double-positive cases, interesting results have been reported with allo-HSCT, which may be a superior therapeutic option (Zanelli et al., 2024). This therapeutic strategy has been adopted in only two cases with concomitant *BCR::ABL1* and *CALR*, and outcome data were available just in one case, which obtained complete remission of both CML and Ph-negative MPN with undetectable genetic markers (Boddu et al., 2018). Due to the limited number of cases, it is difficult to draw any definite conclusion on the optimal treatment modality for patients carrying both *BCR::ABL1* and *CALR*, and data on a larger number of cases are essential to address this issue.

In conclusion, the combination of the *BCR::ABL1* rearrangement and *CALR* mutation is rare but potentially underestimated due to low awareness of this entity, which can impact the therapeutic management and outcome of patients.
