# Supplementary material for: Chronic myeloproliferative neoplasms with concomitant CALR mutation and BCR::ABL1 translocation: diagnostic and therapeutic implications of a rare hybrid disease
Source: Front Cell Dev Biol. 2024 Mar 26;12:1391078. doi: 10.3389/fcell.2024.1391078 (PMC11002177; doi:10.3389/fcell.2024.1391078)
Supplement: Supplementary file 1 [file Table1.DOCX]

Table 1. Cases with coexistent *CALR* mutation and *BCL::ABL1* rearrangement

| **Reference/year** | **Age/sex** | **First diagnosis**  **Clinical manifestations** | **Second diagnosis**  **Clinical manifestations**  **at 2^nd^ disease onset** | **Interval (mos) between 1^st^ and 2^nd^ diagnosis** | **BM histology** | ***BCR::ABL1* and *CALR* clone**  **interaction** | **Type of CALR mutation** | **Therapy/**  **Prognosis/**  **OS since 1^st^ diagnosis** |
| --- | --- | --- | --- | --- | --- | --- | --- | --- |
| Cabagnols  Ref. 17  2015 | 73/F | CML (CP)  At diagnosis, High PTL  high WBC anemia splenomegaly    Under Imatinib: WBC and spleen size reduction, but worsening of anemia and high PTL | MPN, NOS  Worsening of anemia and high PTL  (despite *BCR::ABL1* reduction) | 90 | Grade 2 reticulin fibrosis  no other BM data | Concomitant  *CALR* + *BCR::ABL1+*  *JAK2V617F*- *MPL-*  at CML diagnosis  *CALR-*mutant clone persistent at high allelic burden even at *BCR::ABL1* decrease  (Ph-positive clone sensitive to TKIs, *CALR*-mutant clone persistent) | *CALR* type 1  (52-bp deletion) | Imatinib (with good response of WBC count and spleen size, but worsening of anemia and thrombocytosis despite *BCR::ABL1* decrease); then  Imatinib+ IFN alpha (IFN replaced with HU and EPO for toxic effects); then Imatinib replaced with Dasatinib.  CML relapse due to Dasatinib stopping.  Alive OS 91 mos |
| Loghavi  Ref 18  2015 | 67/M | CML (CP)  High WBC | PMF | 7 | At CML diagnosis:  Hypercellular marrow  myeloid hyperplasia increase of MKs with small and large forms  grade 2 reticulin fibrosis.  (CML+fibrosis+hybrid MKs)  7 mos later (after Dasatinib):  PMF histology  (MKs increase with clustering of large-sized MKs grade 2 reticulin fibrosis+ osteosclerosis) | Concomitant *BCR::ABL1*+78%  *CALR* + 51%  at CML diagnosis;  under Dasatinib:  *BCR::ABL1* 0.21%  *JAK2V617F*-  *CALR* + 68%  (Ph-positive clone sensitive to Dasatinib, *CALR*-mutant clone persistent) | *CALR* type 1  (52-bp deletion) | Dasatinib with DMR and normalization of WBC, but emergence of PMF histology due to *CALR*-mutant clone  Alive OS 10 mos |
| Bonzheim  Ref 19  2015 | 26/M | ET  High PTL | CML (CP)  High WBC  Under Nilotinib, no change in *CALR* allele burden and PTL increase | 40 | At ET diagnosis:  ET histology  Normocellular marrow  Normal M:E ratio  MK increase with hyperlobated forms in loose clusters  No fibrosis.  At CML diagnosis: CML-CP histology  Hypercellular marrow  Increased M:E ratio  MK increase with small hypolobated forms | *CALR* +44%  *JAK2, MPL* and  *BCR::ABL1*- at ET diagnosis;  *BCR::ABL1*+ 99%  *CALR* + 44%  at CML diagnosis  (Ph-positive clone partially sensitive to Nilotinib, *CALR*-mutant clone persistent) | CALR type 2  (5-bp insertion) | IFN with good response, then switched to Nilotinib.  No change in *CALR* allele burden and PTL increase  CML CHR  Alive OS 48 mos |
| Seghatoleslami  Ref 20  2015 | 78/F | CML (BP)+ MPN, NOS  High WBC 18% blasts,  Anemia  high PTL | - | Concurrent diseases | BM aspirate consistent with CML (BP)  BM histology NA | Concomitant *BCR::ABL1*+  *CALR* +  *JAK2 V617F*-  Clone interaction NA | *CALR* type 1  (52-bp deletion) | Therapy and prognosis NA |
| Klairmont  Ref 21  2016 | 90/F | MPN, NOS  Several year history of high PTL | CML (CP)  High WBC  At the time of CML relapse (7 yrs after initial CML),  High WBC with neutrophilia and basophilia anemia and splenomegaly | 468 | At MPN, NOS diagnosis: hypercellular marrow MK hyperplasia  Mild reticulin fibrosis  At CML relapse:  Myeloid hyperplasia atypical MKs severe reticulin fibrosis osteosclerosis  (CML+fibrosis) | At MPN, NOS diagnosis, *BCR::ABL1- JAK2*-  Testing for *CALR* and *MPL* NA  *BCR::ABL1*+51.9% at CML diagnosis;  *BCR::ABL1*+61.72%  *CALR* +  *MPL* mutation + 52% at CML relapse  Clone interaction NA | *CALR* type 1  (52-bp deletion) | HU for high PTL, then Imatinib subsequently switched to Nilotinib  Prognosis NA |
| Diamond  Ref 22  2016 | 50/F | CML (CP)  Anemia mild splenomegaly high WBC | PMF  Anemia, increasing splenomegaly | 48 | At CML diagnosis: severe reticulin fibrosis and hypercellular marrow  (CML+fibrosis)  At PMF diagnosis:  PMF histology  atypical MK hyperplasia and collagen fibrosis | Concomitant *BCR::ABL1*+ 83%  *CALR* +  *JAK2 - MPL- ,* at CML diagnosis.  At PMF diagnosis,  *BCR::ABL1-*  *CALR* + *JAK2- MPL-*  (Ph-positive clone sensitive to Imatinib, *CALR*-mutant clone persistent) | *CALR* type 1  (52-bp deletion) | Imatinib with DMR  Alive OS 60 mos |
| Lewandowski  Ref 23  2017 | 55/F | CML (CP)  High PTL high WBC splenomegaly | MPN, NOS  High PTL despite CML CCyR | 120 | NA | Concomitant *BCR::ABL1*+  *CALR*+ 45%  *JAK2 V617F -MPL*-  at CML diagnosis;  At the time of CML CCyR, *CALR*+ 50%  (Ph-positive clone sensitive to TKIs *CALR*-mutant clone persistent) | NA | HU, IFN+Ara-C, Imatinib, Nilotinib, Dasatinib with CCyR and PTL slight reduction but CALR+ 50%  Alive OS 180 mo |
| Dogliotti  Ref 24  2017 | 61/F | CML (CP) | ET  At ET emergence, high PTL count | 154 | At CML diagnosis, No BM data  ET (or pre-fibrotic PMF) histology at the occurrence of high PTL count | Concomitant *BCR::ABL1*+  *CALR* + 10.5% at  CML diagnosis.  At ET diagnosis,  *BCR::ABL1*-, *JAK2 -CALR* + 46.8%  (Ph-positive clone sensitive to Imatinib, *CALR*-mutant clone persistent with increasing allelic burden after DMR of CML) | *CALR* type 1  (52-bp deletion) | HU, then Imatinib+IFN with CCyR and DMR. Imatinib stopped for gastric intolerance; then enrolment in a peptide CML VAX protocol; DMR at 12 yrs after Imatinib stopping  Alive OS 181 mos |
| Gilles  Ref 25  2017 | 67/F | CML (CP)  At diagnosis, High WBC neutrophilia basophilia | MPN, NOS  High PTL  despite *BCR::ABL1* decrease under Imatinib | 3 | BM consistent with CML, but rarely clustered large MKs with hyperchromatic, multilobated nuclei mild reticulin fibrosis;  (CML+fibrosis+ atypical MKs)  6 months after Imatinib, resolution of CML features, but persistence of atypical MKs | At CML diagnosis*,* concomitant *BCR::ABL1*+ 98.5% and *CALR* + 63%;  After Imatinib *BCR::ABL1* +6.56% and *CALR*+ 52%  (Ph-positive clone sensitive to Imatinib, *CALR*-mutant clone persistent) | *CALR* type 1  (52-bp deletion) | Imatinib with CHR of CML  Alive OS 21 |
| Kandarpa  Ref 26  2017 | 54/M | CML (CP) | PMF  Increasing splenomegaly despite CML DMR | 24 | CML (CP) at CML diagnosis  At PMF diagnosis,  PMF histology  clustering of large MKs with moderate reticulin fibrosis | At CML diagnosis, *BCR::ABL1*+ 17.44%  At PMF diagnosis *BCR::ABL1-* 0.062%  *JAK2-* *CALR*+  Clone interaction NA | NR | Dasatinib with CML DMR  Alive OS 48 mos |
| Kandarpa  Ref 26  2017 | 70/M | PET-MF | CML  Increasing splenomegaly  High WBC anemia | 48 | NR | At PET-MF diagnosis,  *BCR::ABL1, CALR, JAK2, MPL* NP  At CML diagnosis  *BCR::ABL1*+93.3%  *CALR*+  Clone interaction NA | NR | Anagrelide, then Ruxolitinib+ Imatinib (changed to Dasatinib)  CML CHR  Alive OS 60 mos |
| Xia  Ref 27  2018 | 65/F | ET  High PTL | CML (CP)  High WBC with neutrophilia and basophilia anemia | 48 | ET features at marrow aspirate  no BM biopsy at ET diagnosis.  At CML diagnosis, hypercellular marrow increased M:E ratio  hyperplasia of MKs (both small forms with hypolobated nuclei and large forms with hyperlobated nuclei in loose clusters) moderate reticulin fibrosis  (CML+fibrosis+hybrid MKs) | *JAK2-* *BCR::ABL1* –  *CALR* NP at ET diagnosis;  At CML diagnosis  Concomitant *BCR::ABL1+*  *CALR* +  Clone interaction NA | *CALR* type 1  (52-bp deletion) | ASA at ET diagnosis; then Dasatinib; at 20 mos from CML, *BCR::ABL1* decrease (0.45%) persistent high PTL  Alive OS 68 mos |
| Blouet  Ref 28  2018 | 76/M | CML (CP)  High WBC and high PTL | ET  High PTL  despite CML DMR | 3 | BM histology of CML (CP) but rare MKS with large hyperlobated nuclei  (CML+ atypical MKs)  After CML DMR,  ET histology emergence | At CML diagnosis *BCR::ABL1+*  *JAK2 V617F* and *MPL-*  *CALR* + 0.8%  After DMR of CML  *CALR* + 27%  (Ph-positive clone sensitive to Imatinib, *CALR*-mutant clone persistent after DMR of CML) | *CALR* type 1  (52-bp deletion) | Imatinib with DMR but high PTL,  then hydroxycarbamide +Imatinib with PTL decrease; 17 mos after CML diagnosis, CML DMR and good control of ET with CALR+ 25-30%  Alive OS 17 mos |
| Boddu  Ref 29  2018 | 63/F | PMF fibrotic phase  Anemia, mild splenomegaly | CML (CP)  High WBC with neutrophilia and basophilia anemia low PTL hepatomegaly  splenomegaly | 30 mo | At PMF diagnosis, PMF fibrotic phase histology  At CML diagnosis, hypercellular marrow with myeloid hyperplasia and hybrid MKs with small forms and large hyperlobulated forms grade 3 reticulin fibrosis and osteosclerosis  (CML+fibrosis+ hybrid MKs) | At PMF diagnosis,  *BCR::ABL1- JAK2- MPL-* (*CALR* NP)  At CML diagnosis, concomitant  *BCR::ABL1*+ 100%  *CALR* + 45%  Under Imatinib, HU and Ruxolitinib, *BCR::ABL1*+ 56%  *CALR*+ 43%  After allo-SCT, DMR of both diseases | *CALR* type 2  (5-bp insertion) | At PMF diagnosis no therapy  At CML diagnosis HU, ruxolitinib, imatinib with no response  Allo-SCT with complete remission of both diseases and undetectable genetic markers, 2 yrs after allo-SCT  Alive OS 60 mos |
| Boddu  Ref 29  2018 | 65/M | CML (CP) + PMF | - | Concomitant diseases | BM histology at CML complete hematologic response:  hypercellular marrow with myeloid prevalence, atypical large MKs grade 2 fibrosis osteosclerosis | At initial diagnosis, *BCR::ABL1*+ *CALR*+  After CML DMR under Dasatinib, *BCR::ABL1*-  *CALR* + 45-48%;  (Ph-positive clone sensitive to TKI, *CALR*-mutant clone persistent after DMR of CML) | *CALR* type 1  (52-bp deletion) | Dasatinib with DMR and persistence of *CALR* mutation, 2 yrs after diagnosis  CML DMR Alive OS 24 mos |
| De Roeck  Ref 30  2018 | 46/F | ET | CML (CP)  Fatigue abdominal pain high PTL high WBC | 216 | NR | At ET diagnosis, *CALR* NP  At CML diagnosis,  concomitant  *CALR*+  *BCR::ABL1*+  Clone interaction NA | *CALR* type 2  (5-bp insertion) | Ruxolitinb and Imatinib with good control of both diseases  CML DMR  Alive OS 240 |
| Liu  Ref 31  2020 | 46/M | ET  High PTL | CML (CP)  High WBC severe splenomegaly | 132 | At ET diagnosis, ET histology  No reticulin and collagen fibrosis  At CML diagnosis, increased M:E ratio and severe reticulin fibrosis  (CML+fibrosis) | *JAK2*V617F -  *CALR* NP at ET diagnosis  At CML diagnosis, concomitant *BCR::ABL1*+ 111.88%  *CALR* + 37.32%  Under Imatinib, *BCR::ABL1* 0.249% *CALR* 30.60%  (Ph-positive clone sensitive to TKI, *CALR*-mutant clone persistent after CCyR of CML) | *CALR* type 1  (52-bp deletion) | ASA, then IFN with PTL count decrease; then, at CML diagnosis, IFN switched to Imatinib with CCyR 1 yr after CML; at this time PTL increase and *CALR* + 30.60%; then Imatinib +IFN with stable disease  CML DMR  Alive OS 156 mos |
| Da Costa  Ref 32  2020 | 54/M | CML (CP)  High PTL and WBC splenomegaly | ET  PTL increase, despite CML DMR | 120 | At CML diagnosis, high cellularity with high M:E ratio, presence of atypical hyperlobulated MKs  (CML+atypical MKs)  At ET diagnosis,  ET histology | At CML diagnosis, *BCR::ABL1*+  at ET diagnosis, *JAK2- MPL*- *CALR* + *BCR::ABL1-*  Clone interaction NA | *CALR* type 1  (52-bp deletion) | Hydroxycarbamide then Imatinib with DMR 18 mo after CML.  At ET diagnosis, hydroxycarbamide+ ASA with good  PTL count  CML DMR  Alive OS 120 mos |
| Yoon  Ref 33  2020 | 33/F | CML (CP)  High WBC and PTL splenomegaly | ET  Persistent high PTL during TKI despite CML DMR | 51 | At CML diagnosis, hypercellular marrow with MK hyperplasia  Moderate reticulin fibrosis  (CML+fibrosis)  At ET diagnosis, no BM histology | At CML diagnosis, concomitant *BCR::ABL1*+ 25.603%  *CALR* + 10.64% *JAK2* -;  at time of persistent high PTL despite CML DMR, *CALR+* 15.97% *TET2* + 7.83%  (Ph-positive clone sensitive to TKI, *CALR-*mutant clone persistent after DMR of CML) | *CALR* type 1  (52-bp deletion) | Nilotinib with DMR; then (due to persistent high PTL and splenomegaly) Nilotinib changed to Dasatinib+ HU  CML DMR  Alive  OS 51 mo |
| Guidotti  Ref 34  2020 | 80/M | CML (CP)  High WBC High PTL splenomegaly | MPN, NOS  Persistent high PTL during TKI despite CML DMR | 3 | BM histology during TKI therapy:  Hypercellular marrow with increased M:E  Increase of atypical MKs with small hypolobated forms and large hyperlobated in loose clusters mild reticulin fibrosis  (CML+fibrosis+hybrid MKs)  At MPN, NOS diagnosis, no BM histology | At CML diagnosis *BCR::ABL1*+ 81.68%;  Under Imatinib  *BCR::ABL1* 22.31% *CALR* + *JAK2*-  (Ph-positive clone sensitive to TKI, *CALR*-mutant clone persistent) | *CALR* type 1  (52-bp deletion) | Imatinib and ASA, then HU was added for high PTL, then Bosutinib +HU with DMR and normal PTL  Alive  OS ND |
| Balducci  Ref 35  2020 | 73/F | CML (CP)  High WBC  Neutrophilia with 18% myeloid intermediate cells  anemia | ET  High PTL, despite *BCR::ABL1* reduction under Imatinib | 3 | At CML diagnosis, CML (CP) histology  Under Imatinib, ET phenotype emergence | At CML diagnosis, concomitant *BCR::ABL1*+ 91.8%  *CALR* + 46.5%  *JAK2 V617F- MPL -*  Under Imatinib, *BCR::ABL1* 0.01%  *CALR* +48%  (Ph-positive clone sensitive to Imatinib, *CALR*-mutant clone persistent after DMR of CML) | *CALR* type 1  (52-bp deletion) | HU, then Imatinib with *BCR::ABL1* reduction 42% but PTL increase; then Anagrelide+ Imatinib  6 mos later  despite CML DMR *CALR*+ 48% and high PTL,  then Anagrelide switched to IFN +Imatinib with PTL normalization  Alive  OS 26 mos |
| Sobieralski  Ref 36  2022 | 53/F | Prefibrotic PMF, then fibrotic PMF  PTL and LDH increase | CML  WBC increase splenomegaly | 156 | PMF histology at initial diagnosis  At CML diagnosis,  CML +severe reticulin fibrosis | At PMF diagnosis, *BCR::ABL1*- *JAK2*-, 10 yrs after initial diagnosis *CALR* + *ASXL1* exon14+  At CML diagnosis *BCR::ABL1*+ 95%  *CALR* + 92%  ASXL1 exon 14+ 36%  Under Dasatinib,  *BCR::ABL1* decrease 0.516% *CALR*+ 55% *ASXL1*+36%  (Ph-positive clone sensitive to Dasatinib, *CALR*-mutant clone persistent) | *CALR* type 2  (5-bp insertion) | ASA + HU; then ruxolitinib+HU; then Imatinib +Ruxolitinib; then imatinib switched to Dasatinib; spleen RT and allo-HSCT  Prognosis NA |
| Huo  Ref 37  2022 | 42/F | CML  High WBC anemia splenomegaly | PMF  Appetite loss fatigue increased splenomegaly despite CML DMR | 24 | BM aspirate consistent with CML (BM biopsy NP)  At PMF diagnosis, granulocytic and erythroid hyperplasia with severe reticulin fibrosis | At CML diagnosis, *BCR::ABL1*+17.035% *CALR*+34bp deletion 51.3%  At PMF diagnosis *BCR::ABL1*- (0.06%)  *CALR*+ 34bp deletion+ 51.3%  *TET2*+  *MPL- JAK2-*  After Nilotinib+ Ruxolitinib  *BCR::ABL1*- 0.01%  *CALR*+ 46.5%  (Ph-positive clone sensitive to Nilotinib, *CALR*-mutant clone persistent) |  | HU switched to Nilotinib with DMR 12 mo later  At PMF diagnosis, ruxolinitib +Nilotinib with improvement of constitutional symptoms and splenomegaly at 6 mo from ruxolitinib+ nilotinib introduction  DMR of CML with CALR+ 46.5%  Constitutional symptoms absent while splenomegaly and anemia persistent at 4 yrs from PMF diagnosis  Alive OS 70 mos |
| Present case | 45/M | PMF prefibrotic phase | CML  High WBC anemia splenomegaly | 132 | At PMF onset, PMF pre-fibrotic phase histology  At CML diagnosis,  Hypercellular marrow with granulocytic lineage prevalence and hybrid MKs (small forms as well as large hyperchromatic forms with dense clustering)  Reticulin fibrosis grade 2  (CML+fibrosis+hybrid MKs) | At PMF diagnosis, *CALR* + 60% *BCR::ABL1*- *MPL*- *JAK2V617F*-  At CML diagnosis,  *BCR::ABL1*+ 88%  *CALR* + 40%  Under TKI, CML DMR but *CALR* + 40%  (Ph-positive clone sensitive to TKI, *CALR*-mutant clone persistent after DMR of CML) | *CALR* type 1  (52-bp deletion) | Anagrelide, then HU with good control of PMF  At CML diagnosis, Nilotinib+ Ruxolitinib  with CML DMR  At 7 yrs after CML,  Nilotinib+  Anagrelide with DMR  Alive OS 216 mo |

Legends: ASA: acetylsalicylic acid; BM: bone marrow; *CALR*: calreticulin; CHR: complete hematological response; CML: chronic myeloid leukemia; CCyR: complete cytogenetic response; CML (BP): chronic myeloid leukemia (blast phase); CML (CP): chronic myeloid leukemia (chronic phase) DMR: deep molecular remission; EPO: erythropoietin; ET: essential thrombocythemia; F: female; HU: hydroxyurea; IFN: interferon; yrs: years; M: male; M:E: myeloid: erythroid; MKs: megakaryocytes; mos: months; MPN: myeloproliferative neoplasm; MPN, NOS: myeloproliferative neoplasm not otherwise specified; NA: not available; NP: not performed; PMF: primary myelofibrosis; PTL: platelet; TKI: tyrosine kinase inhibitor; VAX: vaccination; WBC: white blood cell
